# Evidence of Selection upon Genomic GC-Content in Bacteria

**DOI:** 10.1371/journal.pgen.1001107

**Published:** 2010-09-09

**Authors:** Falk Hildebrand, Axel Meyer, Adam Eyre-Walker

**Affiliations:** 1 Centre for the Study of Evolution and School of Life Sciences, University of Sussex, Brighton, United Kingdom; 2 Department of Biology, University of Konstanz, Konstanz, Germany; University of Arizona, United States of America

## Abstract

The genomic GC-content of bacteria varies dramatically, from less than 20% to more than 70%. This variation is generally ascribed to differences in the pattern of mutation between bacteria. Here we test this hypothesis by examining patterns of synonymous polymorphism using datasets from 149 bacterial species. We find a large excess of synonymous GC→AT mutations over AT→GC mutations segregating in all but the most AT-rich bacteria, across a broad range of phylogenetically diverse species. We show that the excess of GC→AT mutations is inconsistent with mutation bias, since it would imply that most GC-rich bacteria are declining in GC-content; such a pattern would be unsustainable. We also show that the patterns are probably not due to translational selection or biased gene conversion, because optimal codons tend to be AT-rich, and the excess of GC→AT SNPs is observed in datasets with no evidence of recombination. We therefore conclude that there is selection to increase synonymous GC-content in many species. Since synonymous GC-content is highly correlated to genomic GC-content, we further conclude that there is selection on genomic base composition in many bacteria.

## Author Summary

Shortly after it was proved that DNA was the genetic material it became apparent that organisms, and in particular bacteria, use the four letters of the genetic code to very different extents; the use of G and C varies from less than 20% in some species, to more than 70% in others. This variation in the use of G and C is usually attributed to differences in the pattern of mutation between species. Here we test this hypothesis, and show that, on the contrary, there seems to be pervasive selection on the base composition of the bacterial genome, particularly in GC-rich species. This suggests that many, if not all, sites may be subject to natural selection in many bacteria. Unfortunately, the reason why some bacteria should be selected for high GC-content remains unclear.

## Introduction

Bacteria show an astonishing diversity of genomic GC-contents, from species such as the endosymbiont *Carsonella ruddii*, which has a GC-content of 16.5% [1], to *Anaeromyxobacter dehalogens*, which has a GC-content of 75%. This is particularly surprising given that bacteria generally have little intergenic DNA, so the extreme variation in base composition must affect both coding and non-coding sites; this is evident in the correlation between the GC-content of the first two codon positions of protein coding genes, and genomic GC-content [2]–[4] (Figure S1). The variation in base composition is not restricted to particular groups of bacteria. Although, genomic GC content shows a fair level of phylogenetic inertia [5], most classes of bacteria show substantial variation in genomic GC content; one of the most dramatic examples is afforded by the α-proteobacteria, which have genomic GC-contents ranging from less than 30% to greater than 60% [6].

The reasons for the variation in genomic GC-content are controversial. In the first example of a “neutral” theory being used to explain a phenomenon at the molecular level, Sueoka [7] and Freese [8] independently suggested that the extreme variation in genomic base composition between bacteria was a consequence of differences in the mutation pattern. Fifty years on and there is little evidence to contradict this view. It has been shown that genomic GC-content is correlated with a number of factors including genome size [6], whether the bacterium is free-living or not [9], [10], the environment [11], aerobiosis [12], nitrogen utilization [13] and possibly temperature [14] (but see [15]–[17]). However, it is still unknown whether these correlations are due to the influence of these factors on the process of mutation, or whether natural selection is involved.

For a few bacteria there is evidence that the genomic GC-content is not a simple consequence of mutation bias. The mutation pattern has been directly measured in *Escherichia coli* and found to be AT-biased [18], [19]. If the genomic GC-content was simply determined by mutation bias it would be expected to decline from its current value of 0.5 to 0.32 [20]. The fact that the genomic GC-content is higher than this value can be explained by functional constraints. However, even sites that appear to be under little selection, such as synonymous sites in lowly expressed genes, have a GC-content of ∼57% [21], which is considerably above the equilibrium inferred from the mutation studies. Of course, it is likely that the mutation pattern measured in the laboratory is not identical to that experienced by the bacterium in the wild. However, a phylogenetic analysis of substitutions between closely related *E.coli* strains also shows the same tendency for an excess of GC→AT mutations [22]. Furthermore this bias towards GC→AT mutations is less acute amongst common polymorphisms, consistent with natural selection acting against GC→AT mutations [22].

A similar excess of GC→AT substitutions is seen in the pseudogenes between strains of *Mycobacterium leprae* [23], again suggesting that the mutation pattern is AT biased relative to the current genomic GC-content, and that it will, given the opportunity, decrease the GC-content [20]. However, *M. leprae* is an intra-cellular parasite and this has led to the loss of many genes; it is possible that one of these is a DNA repair enzyme that has shifted the mutation bias towards being AT-rich.

Here we test whether genomic GC-content is a simple consequence of mutation bias by investigating the pattern of synonymous genetic variation in 149 bacterial species.

## Results

To investigate whether genomic GC-content is solely a consequence of mutation bias, we analysed the pattern of synonymous polymorphism at the third position of 4-fold degenerate codons. Since, the GC-content of 4-fold sites (GC4) is strongly correlated to genomic GC-content [2]–[4] (Figure S1) we expect many of the forces that act upon synonymous sites to also act upon the genome as a whole. If GC4 is simply a consequence of mutation bias and the base composition is at equilibrium, then we expect equal numbers of GC→AT and AT→GC synonymous mutations at 4-fold sites to be segregating within a species [24], [25]. To test this prediction we assembled polymorphism datasets from 149 bacterial species from 8 phyla, 15 classes and 77 genera, in which we had at least 8 sequences from species members, 10 or more synonymous polymorphisms at 4-fold degenerate sites and the nucleotide diversity at four-fold sites for GC↔AT mutations was less than 0.1 (see Table S1 for a full list of species).

The concept of a species is potentially problematic in bacteria because they do not undergo conventional sexual reproduction. However, “population genetic” species do exist, in the sense that strains exist that undergo random genetic drift and selection together [26]. Unfortunately, it is not possible to determine which strains meet this criterion. A named bacterial species may include two groups of strains that are effectively two different population genetic species. As a consequence some single nucleotide polymorphisms (SNPs) may be substitutions between species. However, this should not affect our results in a consistent direction.

Ideally we would infer the pattern of mutation from our SNPs within a likelihood framework, integrating across all possible phylogenetic trees and ancestral states. However, this approach was not possible because a closely related outgroup was not available for most datasets. We therefore used two alternative methods to infer the direction of mutation. In the first we used the allele frequencies, inferring the minor allele to be the new mutation; we also reconstructed the phylogenetic tree between strains for each gene and used parsimony to infer the ancestral state and hence the direction of mutation. These two approaches gave qualitatively similar results, but we present the results from the frequency method because the potential biases are easier to estimate; simulations suggest that parsimony typically outperforms the frequency method, but the biases are less easy to predict. We restrict the analysis to datasets in which synonymous diversity was less 0.1 for two reasons; first to concentrate the analysis on strains that are likely to form a species, in the sense that they undergo selection and random genetic drift together, and second, to limit problems with violation of the infinite sites assumption (the assumption that each mutation is fixed or lost at a site before the next one occurs).

Overall we observe a large excess of GC→AT mutations at 4-fold sites (11045 GC→AT versus 8309 AT→GC, p<0.0001 using a two-tail binomial test), with similar patterns evident at 2-fold sites (6282 GC→AT and 5196 AT→GC p<0.0001) (Table S1). The patterns we observe are consistent with measurements of the mutation pattern in *E. coli* which has been studied directly using reporter constructs [18], [19] or by phylogenetic reconstruction [22]. The bias is slightly more extreme in the reporter constructs (273 GC→AT versus 131 AT→GC, ratio = 2.1) [18], [19] than in our analysis (1223 GC→AT versus 647 AT→GC, ratio = 1.9) but this is to be expected since our method will misinfer the direction of some mutations (about 20% of neutral mutations in a stationary population for the level of diversity and GC-content observed in *E. coli*).

**Figure 1:**
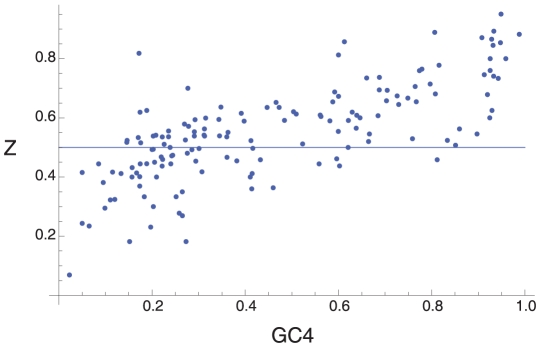
The pattern of synonymous SNPs. The figure shows the correlation between Z, the proportion of GC↔AT SNPs that are GC→AT, and GC4, across 149 bacterial species.

The proportion of GC↔AT mutations that are GC→AT, *Z*, is strongly correlated to GC4 (Figure 1 and S2; r = 0.73, p<0.0001) such that species with high GC4 have a large excess of GC→AT changes while species with low GC4 have an excess of AT→GC mutations. There is evidence that GC-rich and GC-poor species behave qualitatively differently since the regression line intercepts the Z = 0.5 line at a GC4 value that is significantly different to zero or one; the point of intercept is 0.34 with 95% CIs 0.29, 0.38. Henceforth we define GC-rich and AT-rich species relative to this point of interception. In doing so, we find that 69 of 82 (84%) of the GC-rich species have an excess of GC→AT (i.e. Z>0.5) (two tail binomial test, p<0.0001), and 42 of 67 (63%) of AT-rich species have Z<0.5 (p = 0.050). These patterns are not restricted to particular phylogenetic groups; they are found, with only two exceptions, across all phyla and classes that we have investigated (Table 1); the exceptions involve two classes that contain very few species and thus provide little data.

**Table 1:** The mean value of Z across bacterial phyla and classes.

Phylum	Class	No. of species	GC4 range	Mean Z (GC4<0.34)	Mean Z (GC4>0.34)
Actinobacteria	Actinobacteria	3	0.64–0.93	no species	0.64
Bacteroidetes/chlorobi	Bacteroidetes	3	0.12–0.46	0.43	0.36
Chlamydiae/verrucomicrobia	Chlamydiae	2	0.21–0.30	0.45	no species
Cyanobacteria	Chroococcales	2	0.38–0.51	no species	0.53
Cyanobacteria	Nostocales	3	0.26–0.31	0.45	no species
Cyanobacteria	Oscillatoriales	2	0.41	no species	0.38
Cyanobacteria	Stigonemales	1	0.40	no species	0.59
Firmicutes	Bacilli	27	0.085–0.68	0.44	0.58
Firmicutes	Clostridia	5	0.050–0.28	0.34	no species
Proteobacteria	Alphaproteobacteria	16	0.099–0.94	0.43	0.65
Proteobacteria	Betaproteobacteria	6	0.66–0.96	no species	0.67
Proteobacteria	delta/epsilon subdivisions	6	0.15–0.99	0.49	0.78
Proteobacteria	Gammaproteobacteria	62	0.095–0.95	0.50	0.66
Spirochaetes	Spirochaetes	7	0.12–0.60	0.45	0.54
Tenericutes	Mollicutes	4	0.023–0.24	0.33	no species

The excess of GC→AT mutations in GC-rich species and the excess of AT→GC mutations in AT-rich species could potentially be due to sequencing error or a violation of the infinite sites assumption. The infinite sites assumption is important for the following reason. Let us imagine that we have a GC-rich species in which high GC content is a consequence of mutation bias. This implies that AT nucleotides are more mutable than GC nucleotides, but when mutation rates are low, such that all mutations occur at sites which are monomorphic, we expect on average to observe equal numbers of GC→AT and AT→GC mutations [25]. However, as the mutation rate increases, so mutations sometimes occur at sites that already have the same mutation segregating; these new mutations are therefore less likely to generate a new observed polymorphism – for example the mutation may already be at such a high frequency that it is very likely to be observed in a sample of sequences. This violation occurs more readily for AT→GC mutations, because their rate of mutation is higher. Thus as the overall mutation rate increases, so an excess of GC→AT mutations is generated.

**Figure 2:**
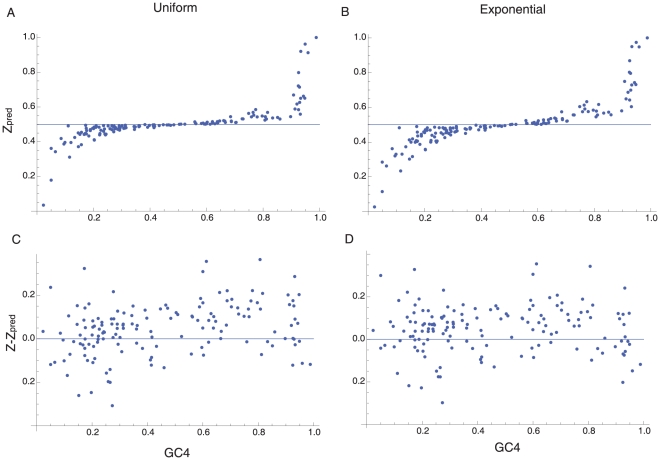
The infinite sites assumption. The figure shows the predicted value of Z, allowing for a violation of the infinite sites assumption, assuming that base composition is due to mutation bias alone and base composition is stationary, plotted against GC4 under the (A) constant rate and (B) exponential rate models, along with the effect of removing this bias from the observed value (Z-Z_pred_) for the (C) constant and (D) exponential models.

However, several lines of evidence suggest that violation of the infinite sites assumption is not responsible for the biases in SNPs that we observe. First, we note that the frequency method will be unbiased under the mutation bias hypothesis when base composition is stationary and the GC-content is 50%, whether or not there is a violation of the infinite sites assumption: in 6 out 7 species with GC4 between 0.45 and 0.55 Z>0.5 (p = 0.13) and there are 770 GC→AT and 529 AT→GC mutations in these species (p<0.0001). Second, we note that if we restrict the data to singletons, which are more likely to reflect the pattern of mutation, we find a large excess of GC→AT mutations in GC-rich species and the opposite pattern in AT-rich species: Z>0.5 in 69 out 82 GC-rich species (p<0.0001), and Z<0.5 in 47 of 67 AT-rich species (p = 0.001). However, to further investigate whether the biases could be due to the infinite sites assumption we used population genetic theory to predict the value of Z, allowing a violation of the infinite sites assumption (see Materials and Methods). For each species we determined the mutation rate required to generate the observed level of nucleotide diversity at 4-fold sites for GC↔AT mutations and then used this to predict Z under the assumption that the base composition is stationary and determined by mutation bias alone. If we assume the mutation rate is constant across sites we find that Z is expected to be close to 0.5 when the GC-content is between 30 and 70%, but that it can be substantially biased outside these limits (Figure 2A). However, Z is typically greater than Z_pred_ in GC-rich genes, even in very GC-rich species that have the highest bias (Figure 2C): in 74% of GC-rich species Z>Z_pred_ (p<0.0001). In contrast, in only 43% of AT-rich species is Z<Z_pred_ (p = 0.33).

The mutation rate is known to differ between sites in bacteria so we also investigated a model in which the mutation rate was exponentially distributed across sites. An exponential distribution of rates represents substantial variation in the mutation rate: the mutation rate of the 95^th^ percentile is ∼60-fold higher than the 5^th^ percentile, the 99^th^ percentile is ∼460 fold higher than the 1^st^ percentile. As expected, under an exponential distribution the biases in Z_pred_ are more extreme than under a constant rate model (Figure 2B). Nevertheless, Z>Z_pred_ in 68% of GC-rich species (p<0.0012) (Figure 2D). In AT-rich species only 31% of species show Z<Z_pred_; this is significant (p = 0.003) but in the wrong direction suggesting that we may be over-correcting for violations of the infinite sites assumption, possibly because we have assumed too much variation in the mutation rate. It thus seems that GC-rich bacteria have a genuine excess of GC→AT mutations. In contrast, the excess of AT→GC mutations in AT-rich species may be due to a violation of the infinite sites assumption. Consistent with this, we find that approximately half of species have Z<0.5 if we restrict our analysis to species with low synonymous site diversity, which reduces violations of the infinite sites assumption: 14 out of 25 species for a diversity of 0.03, and 5 out of 11 for a diversity of 0.02. Inferring the direction of mutation using parsimony also shows an excess of AT→GC mutations in AT-rich species (Figure S1), and this method appears to be more robust than the frequency method. However, parsimony can be biased when there is a high mutation rate and strong base composition bias [27], so it is not currently possible to rule out a violation of the infinite sites assumption as the reason for the excess of AT→GC SNPs in AT-rich species. In what follows, we largely concentrate on the results from the GC-rich species, since the excess of GC→AT mutations in GC-rich species appears to be genuine, but the excess of AT-GC mutations in AT-rich species may not be.

The excess of GC→AT mutations in GC-rich species does not seem to be due to sequencing error since the results remain qualitatively unaffected by the removal of singletons: Z>0.5 for 73% of GC-rich species (p<0.0001).

**Figure 3:**
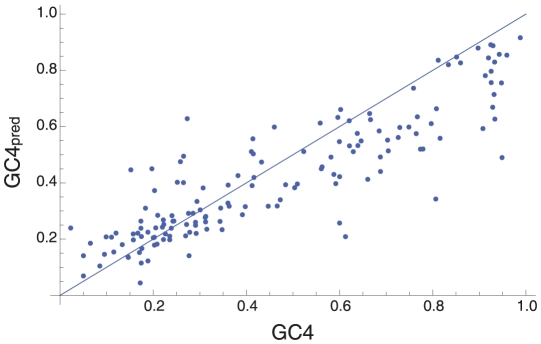
The equilibrium GC content under the mutation bias model. The figure shows the relationship between GC4_pred_, the GC4 to which each species is predicted to evolve under mutation pressure, and the current GC4.

The pattern of SNPs implies, assuming that GC4 is determined by mutation bias alone, that most GC-rich species are declining in GC4. This can be illustrated by using the observed numbers of GC→AT and AT→GC mutations to predict the GC4 value, GC4_pred_, to which each species would evolve under mutation bias if there was no selection (Figures 3 and S3). The predicted GC4 is lower than the current GC4 for most GC-rich species; in many cases the difference between the current GC4 and the predicted GC4 is underestimated because the direction of some SNPs will be misinferred. Such a general decrease in GC-content across GC-rich species is clearly unsustainable, as it would lead to a great reduction in the variance in GC4 over time. This therefore suggests that selection, or some other force, is maintaining high GC4 in many bacteria.

**Figure 4:**
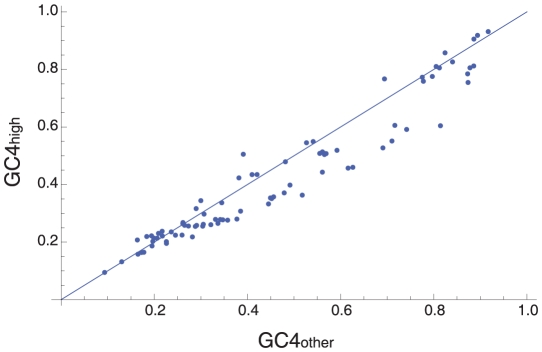
Translational selection. The figure shows the relationship between GC4 for putatively highly expressed genes and GC4 for all other annotated genes. The line is for GC4_high_ = GC4_other_.

It is well known that selection acts upon synonymous codon use in bacteria to increase translational efficiency [28] and this could be maintaining high or low GC4. To investigate this we determined the GC4 for the genes that are generally highly expressed (ribosomal proteins and elongation factors Tu and Ts) and subject to strong translational selection [4], GC4_high_, in each of the 84 species for which a genomic sequence was available in our analysis. We compared this to the GC4 of all other protein coding genes, GC4_other_, from the genome. In most species GC4_high_ is lower than GC4_other_, suggesting that selection on translational efficiency tends to favour lower GC4 (Figure 4). Thus translational selection cannot explain the high GC-content, or the patterns of polymorphism we infer. If we restrict our analysis to those GC-rich species in which GC4_high_<GC4_other_ we find that there is an excess of GC→AT mutations in GC-rich species: Z>0.5 in 29 out of 31 (94%) of GC-rich species (p<0.0001) and Z>Z_pred_ in 84% (p = 0.0002) and 77% (p = 0.0033) of GC-rich species under the constant and exponential models respectively. However, in almost all AT-rich species GC4_high_<GC4_other_, so we cannot exclude the possibility that the excess of AT→GC SNPs in these species is due to translational selection, if it is not due to a violation of the infinite assumption.

The base composition of many eukaryotes is thought to be affected by biased gene conversion (BGC) [29]–[31], and since prokaryotes can undergo recombination and have a GC biased repair system [29], it is likely that they can also undergo BGC. This might therefore explain the excess of GC→AT mutations, since BGC acts, at a population genetic level, like selection [32], [33]. To investigate this hypothesis we used two approaches. First, we removed all datasets that had evidence of recombination, gene conversion or BGC by removing datasets in which all four haplotypes were present for any pair of bi-allelic sites – the so called four gamete test [34]. We used this relative simple method because it is unclear how methods designed to estimate gene conversion behave when conversion is biased. We find that for the 60 species for which we have data, Z>0.5 for 19 of 28 GC-rich species (p = 0.087) and that these overall contain 1079 GC→AT and 844 AT→GC mutations (p<0.0001). Second, we tested whether GC4, Z and Z-Z_pred_ were correlated to the rate of recombination. Vos and Didelot [35] have recently estimated a quantity correlated to the rate of recombination - this is the probability that a nucleotide will change through recombination relative to the probability that it will change through mutation (r/m) - in 48 bacterial species, of which 34 are represented in our dataset. We find no evidence that r/m is correlated to GC4 (r = −0.076, p = 0.67), Z (r = 0.003, p = 0.99) or Z-Z_pred_ (uniform rate model: r = 0.026, p = 0.88; exponential rate model: r = 0.017, p = 0.92). However, biased gene conversion depends on the rate of recombination, multiplied by the effective population size [32], rather than relative to the mutation rate. We therefore multiplied r/m by the synonymous nucleotide diversity for 4-fold GC↔AT mutations to yield a statistic which is likely to be correlated to 2*N_e_q*, where *q* is the rate of recombination per nucleotide and *N_e_* is the effective population size. There is no evidence that this new statistic is correlated to GC4 (r = 0.039, p = 0.83), Z (r = 0.11, p = 0.55) or Z-Z_pred_ (uniform rate model: r = 0.18, p = 0.32; exponential rate model: r = 0.18, p = 0.30). There is therefore no evidence that the excess of GC→AT mutations is a consequence of biased gene conversion.

Bacteria can undergo horizontal gene transfer (HGT) in which a gene, or gene fragment, from a distantly related species can be incorporated into the genome [36]. This can involve two separate processes: the gene or gene fragment can be incorporated into a pre-existing gene by homologous recombination, or it may represent a new gene. We refer to these processes as homologous HGT, hHGT, and non-homologous HGT, nhHGT. Most genes that are transferred via nhHGT tend to be AT-rich [37], [38]. As such we would expect an excess of AT→GC SNPs, under the mutation hypothesis, as the gene evolves to the GC-content of the host [39]. This is opposite to the pattern we observe. However, to investigate the matter further, we restricted the analysis to a set of genes that appear to rarely undergo nhHGT as specified by Bern and Goldberg [40]; the genes were mapped to this list using the gene name. We find that Z>0.5 in 37 out of 40 GC-rich species for which we still have sufficient data (10 or more 4-fold degenerate synonymous polymorphisms) (p<0.0001), and that Z>Z_pred_ in 80% (p = 0.0002) and 70% (p = 0.017) of GC-rich species using the uniform and exponential models respectively.

In contrast to nhHGT, hHGT could explain the excess of GC→AT SNPs in GC-rich species. It is likely that many gene or gene fragments transferred by hHGT will be less extreme in GC-content than the genome they integrate into if the genome is GC-rich. The introduced sequence may therefore generate a series of GC→AT SNPs. This situation will be temporary because either the new sequence will be lost, or it will become fixed. If it becomes fixed, it will then evolve to the GC-content of its new host, in the process generating an excess of AT→GC SNPs under the mutation bias hypothesis. Thus an excess of GC→AT SNPs can be only be generated if AT-rich sequences are continually introduced by hHGT and then lost. This process would have to be pervasive to explain our results, affecting the majority of GC-rich species and generating most of the SNPs within them. This seems unlikely. However, to investigate the matter further we used Maynard Smith's *maxchi* test [41] to exclude datasets in which part of a gene has recently undergone hHGT and is polymorphic within the population: Z>0.5 in 33 (80%) of 41 GC-rich species for which we still sufficient data once we have removed those datasets with evidence of HGT (p = 0.0001) and Z>Z_pred_ in 68% (p = 0.027) and 66% (p = 0.060) of GC-rich species under the constant and exponential models respectively.

## Discussion

**Figure 5:**
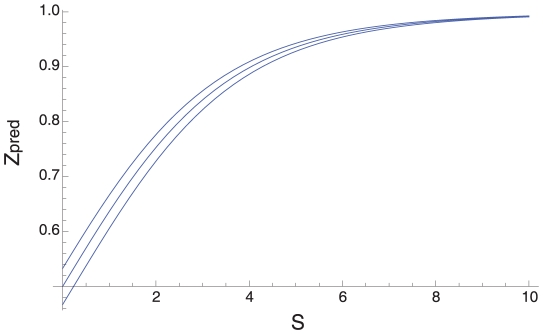
Selection on GC-content. The figure shows the effect of selection in favour of GC on *Z*. The relationship between *Z_pred_* and *S* (2*N_e_s*) for three values of *f*, the equilibrium GC-content when there is no selection (i.e. the mutation bias); from top to bottom *f* is 0.7, 0.5 and 0.3. In these examples 2*N_e_μ* = 0.1 and *n* = 10; qualitatively similar patterns are observed for other values of these two parameters.

We have shown that there is a large excess of GC→AT synonymous SNPs segregating at 4-fold degenerate sites in GC-rich bacteria, with AT-rich bacteria showing the opposite pattern. These patterns are found across different phyla and classes of bacteria suggesting that these patterns are not restricted to select groups of bacteria. We have shown that the excess in GC-rich bacteria is probably not due to sequencing error, a violation of the infinite sites assumption, translational selection, biased gene conversion or horizontal gene transfer. In contrast the excess of AT→GC SNPs in AT-rich species may be due to either a violation of the infinite sites assumption, translational selection, or selection for low GC-content. The excess of GC→AT SNPs in GC-rich species is consistent with selection [24]; under selection in favour of increased GC we expect to see an excess of GC→AT SNPs, even when the direction is inferred from the allele frequency (Figure 5). We therefore conclude that there is selection to increase GC4 in GC-rich species. Since there is a strong correlation between GC4 and genomic GC-content [2], [4] (Figure S1) we furthermore infer that selection is acting to increase not only GC4, but also the genomic GC-content in GC-rich species, as others have suggested before based on less extensive data [20], [22].

Our results are in accord with those in an accompanying paper in this journal by Hershberg and Petrov [42]. They have investigated the pattern of mutation in five clonal pathogens using a phylogenetic analysis of closely related strains and an outgroup. They infer the pattern of mutation to be AT biased in all species, even those that are GC-rich. This suggests that there is a universal mutation bias towards AT, as Lynch has suggested previously based on more limited data [43], [44]. This in turn suggests that there must be some pressure maintaining high GC content in those species that have a high genomic GC content.

We have investigated whether the bias towards GC→AT SNPs, in GC-rich species, is due to biased gene conversion by removing all datasets which fail the four gamete test, and testing whether GC-content and the bias towards GC→AT SNPs is correlated to measures of recombination. Biased gene conversion is a process that drives mutations through a population; since it is not expected to affect the frequency of mutations at linked sites it is expected to generate four gametes during this process. Nevertheless the FGT may miss some datasets that are undergoing gene conversion and we cannot completely rule out biased gene conversion as an explanation. Intriguingly, it has recently been shown that the GC-content across the *E.coli* genome is correlated to the rate of recombination; this is consistent with biased gene conversion [45]. However, both the rate of recombination and GC content are correlated to the distance from the origin and terminus of replication, so it is unclear whether there is a causal relationship between the two.

It has been suggested that there is a universal mutational bias in both prokaryotes and eukaryotes towards AT [42]–[44]. Our analysis provides some limited support for this hypothesis. To a first approximation we can infer whether the mutation pattern is AT-biased by assuming there is no selection and estimating the GC4 to which the sequences would evolve, an AT-biased mutation pattern being one that will give an equilibrium GC4<0.5. This method is slightly liberal, because under selection there will be an excess of deleterious mutations segregating which will slightly exaggerate the apparent mutation bias. Almost all species with GC4<0.6 are predicted to evolve to GC4<0.5 if selection was relaxed (Figure 3), however for many species with GC4>0.6 they are predicted to evolve a lower GC4, but not one which would indicate an AT-biased mutation pattern. This may be because, as the true bias increases, so the level of mis-inference increases. This can be seen by considering an extreme example; imagine that all mutations are GC→AT and the predicted GC4 is therefore zero. Some of the SNPs will be mis-inferred as AT→GC and hence the predicted GC4 will be greater than zero. The bias towards AT seems to be largely due to a bias in transitions; across the whole dataset there are 9162 GC→AT and 6694 AT→GC transitions at 4-fold degenerate sites, and 1883 GC→TA and 1615 TA→GC transversions.

Endosymbiotic bacteria typically have low AT-contents [46]. This is often ascribed to a loss of DNA repair genes due to their small effective population size and strict clonality, which leads to the accumulation of deleterious mutations [46], although selective explanations have also been proposed [9]. However, the low GC content of endosymbionts might in part be due to a more direct effect of their small effective population size [22], [42]; there might be selection for increased GC content but this is ineffective in endosymbionts, leading to low GC content. In support of this hypothesis, the pattern of substitution between and within *Buchnera* species suggests that they are equilibrium and that there is no selection acting upon base composition [47], [48] (Table S1).

Although, most obligate endosymbionts have low GC content, *Candidatus Hodgkinia cicadicola* is an exception with a genomic GC content of 58% [49]. This is a challenge to any selective explanation of GC-content since selection appears to be less effective in endosymbionts because of their small effective population size [10], [46]. There are a number of possible explanations. First, it might be that selection is sufficiently strong to overcome the decreased efficiency of selection. Second, it might be that a mutation conferring a GC-biased mutation pattern has fixed in *Hodgkinia*. Neither of these explanations is very satisfactory and it will be of great interest to see what pattern of SNPs is present within this species.

Although, we have evidence of selection on GC-content in GC-rich bacteria, the nature of the selective agent is unclear. Recently Foerstner et al. [11] showed that bacterial communities from particular environments have surprisingly narrow GC-content distributions and that these distributions differ between environments; for example the bacteria from a sample of surface seawater had a median GC-content of 34%, while a soil sample had a median of 61%. This difference was not due to different phyla being present in the different environments. This suggests, in association with our results, that certain GC-contents are favoured in particular environments. But why this is so, remains a mystery.

## Materials and Methods

The Popset database of Genbank was searched for the keyword “bacteria”. From this we extracted datasets in which we had at least 8 sequences from the same species, defined as a group of bacterial strains with the same species and genus name. These sequences were translated, aligned using MUSCLE [50] and back translated to DNA. We inferred the direction of mutation using two methods. In the first we used the allele frequencies inferring the minor allele to be the new mutation; sites with more than two alleles, or two alleles at equal frequency were discarded. In the second method, we reconstructed the phylogenetic tree between strains using minimum evolution as implemented in FastME [51], rooted the tree assuming a molecular clock and then used parsimony to infer the ancestral state. We only analysed species for which we had at least 10 synonymous GC↔AT single nucleotide polymorphisms (SNPs) segregating at 4-fold degenerate sites. To estimate the confidence intervals for the GC4 value at which the regression line intercepted the Z = 0.5 or Z-Z_pred_ = 0 lines we bootstrapped the data by species. We inferred the GC-content to which a sequence would evolve under mutation bias from the current GC4 and the numbers of GC→AT SNPs, *U*, and AT→GC SNPs, *V* as
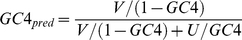
(1)A similar equation allows one to infer the predicted GC-content at 2-fold sites. To detect possible cases of horizontal gene transfer we ran the *maxchi* test [41] with a slight adjustment to improve sensitivity as suggested by [52].

### Theory

Using population genetic theory we can infer the expected proportion of SNPs that are GC→AT, Z, under models in which the base composition is determined by mutation bias alone, and in which there is both selection and mutation bias acting. The direction of mutation is assumed to be inferred from the allele frequencies. We only consider changes between GC and AT so the system is effectively biallelic. Let the mutation rate from GC→AT be *u* and let the mutation rate from AT→GC be *v*. In the case of the selection model let GC mutations have an advantage of +*s* over AT alleles. We will assume the organism is haploid and the population is stationary in size. Under these assumptions the distribution of the frequency of GC at a site, *x*, or equivalently the frequency of GC across many identical sites, is
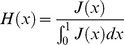
(1)where
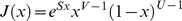

*U* = 2*N_e_μ(1-f)*, *V* = 2*N_e_μf*, *S* = 2*N_e_s*, *μ* is a mutation rate constant and *f* = *v/(u+v)* [53]. If *U*<<*1* and *V*<<1 then *f* is the mean frequency of GC across sites, or the time the site is monomorphic for the GC allele. If we sample *n* chromosomes from this population the probability of detecting *i* instances of GC is
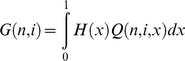
(2)where
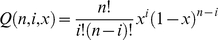
From equation 2 it is straightforward to calculate the numbers of SNPs inferred to be GC→AT and AT→GC
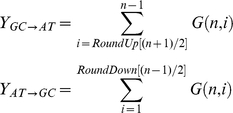
(3)and hence the proportion to be GC→AT is
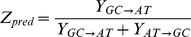
(4)We can also calculate the nucleotide diversity
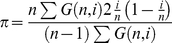
(5)Simple extensions of the above equations allow one to have a distribution of mutation rates across sites by integrating across the required distribution. We considered an exponential distribution.

In Figure S4 Z_pred_ is plotted against the nucleotide diversity when there is no selection for values of *f*, where *f* is the equilibrium GC-content under infinite sites assumption; we only consider *f*>0.5 because the system is symmetrical. Under the null hypothesis we expect Z_pred_ = 0.5, and this expectation is observed when *f*<0.7. However, Z can be substantially greater than 0.5 when *f*>0.7 and the nucleotide diversity is large; this bias arises because of a violation of the infinite sites assumption. However, these biases are small when the diversity is less than 0.02. In Figures 2A and 2B we plot Z_pred_ for each dataset considered in our analysis given the observed nucleotide diversity; Z_pred_ is mildly dependent upon the number of strains that have been sequenced and so we take the median value if multiple genes have been sequenced in a species.

We can also use the equations above to demonstrate that Z_pred_ is expected to be generally greater than 0.5 if selection favours GC irrespective of the mutation bias (and since the system is symmetrical we expect Z<0.5 when selection favours AT) (Figure 5). However, it can also be less than 0.5 if the mutation pattern is biased in favour of AT and selection for increased GC is weak or absent, but this bias is generally small.

## Supporting Information

Figure S1 GC content correlations in prokaryotes. Figure shows the GC-content of the (A) first two and (B) third codon positions versus genomic GC-content for 855 complete bacterial genomes.
(0.28 MB EPS)

Figure S2 Using parsimony to infer the direction of SNPs. Figure shows the relationship between the proportion of GC↔AT SNPs that are GC→AT, Z, and GC4, where the direction of a SNP is inferred by parsimony. The line is where Z = 0.5.
(0.21 MB PDF)

Figure S3 The equilibrium GC content under the mutation bias model. The figure shows the relationship between GC4_pred_, the GC4 to which each species is predicted to evolve under mutation pressure from SNPs inferred by parsimony, and the current GC4.
(0.28 MB EPS)

Figure S4 Violation of the infinite sites assumption. The figure shows the relationship between Z_pred_ and π_s_ under the neutral equilibrium model for different equilibrium GC-contents for (A) 8 strains and (B) 50 strains, when violation of the infinite sites assumption is allowed. The lines from left to right are for f = 0.95, 0.9, 0.8, 0.7, 0.6.
(0.30 MB EPS)

Table S1 The species analysed along with their phylum, class, the numbers of GC→AT and AT→GC at 4-fold sites (U4 and V4 respectively) and 2-fold (U2 and V2 respectively) sites, Z, GC4 and the GC4 to which the sequence is predicted to evolve under mutation bias alone, GC2 the GC-content of 2-fold sites and Z_pred_ under the uniform and exponential models respectively. Also included is the nucleotide diversity for GC↔AT mutations at 4-fold sites and the genomic GC4 of highly expressed and other genes.
(0.11 MB XLS)
