# Supplementary figures and images for: Evidence of Selection upon Genomic GC-Content in Bacteria
Source: PLoS Genet. 2010 Sep 9;6(9):e1001107. doi: 10.1371/journal.pgen.1001107 (PMC2936529; doi:10.1371/journal.pgen.1001107)

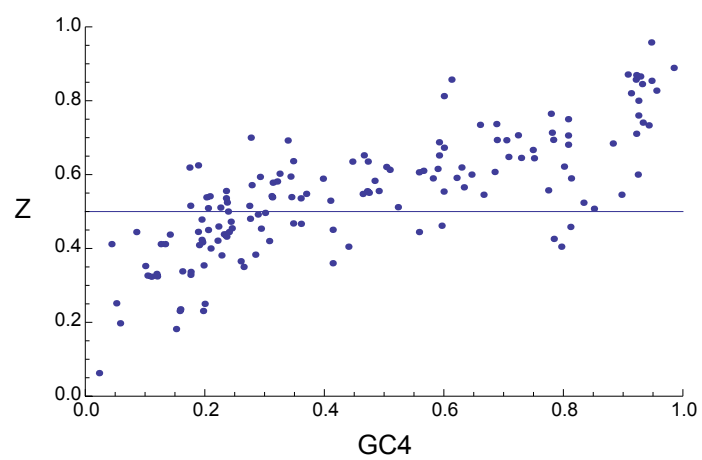

Supplement: Figure S2 — Using parsimony to infer the direction of SNPs. Figure shows the relationship between the proportion of GC↔AT SNPs that are GC→AT, Z, and GC4, where the direction of a SNP is inferred by parsimony. The line is where Z = 0.5. (0.21 MB PDF) [file pgen.1001107.s002.pdf]
